# Fabrication of Polydimethysiloxane (PDMS) Dense Layer on Polyetherimide (PEI) Hollow Fiber Support for the Efficient CO_2_/N_2_ Separation Membranes

**DOI:** 10.3390/polym13050756

**Published:** 2021-02-28

**Authors:** Guoqiang Li, Katarzyna Knozowska, Joanna Kujawa, Andrius Tonkonogovas, Arūnas Stankevičius, Wojciech Kujawski

**Affiliations:** 1 Faculty of Chemistry, Nicolaus Copernicus University in Toruń, 7, Gagarina Street, 87-100 Toruń, Poland; grantli@doktorant.umk.pl (G.L.); katkno@doktorant.umk.pl (K.K.); joanna.kujawa@umk.pl (J.K.); 2 Lithuanian Energy Institute, 3, Breslaujos Street, LT-44403 Kaunas, Lithuania; Andrius.Tonkonogovas@lei.lt (A.T.); Arunas.Stankevicius@lei.lt (A.S.); 3 National Research Nuclear University MEPhI, 31, Kashira Hwy, 115409 Moscow, Russia

**Keywords:** polyetherimide (PEI) hollow-fiber support, thin film composite membranes, dip-coating conditions, polydimethysiloxane (PDMS) dense layer, gas separation

## Abstract

The development of thin layer on hollow-fiber substrate has drawn great attention in the gas-separation process. In this work, polydimethysiloxane (PDMS)/polyetherimide (PEI) hollow-fiber membranes were prepared by using the dip-coating method. The prepared membranes were characterized by Scanning Electron Microscope (SEM), energy-dispersive X-ray spectroscopy (EDX), and gas permeance measurements. The concentration of PDMS solution and coating time revealed an important influence on the gas permeance and the thickness of the PDMS layer. It was confirmed from the SEM and EDX results that the PDMS layer’s thickness and the atomic content of silicon in the selective layer increased with the growth in coating time and the concentration of PDMS solution. The composite hollow-fiber membrane prepared from 15 wt% PDMS solution at 10 min coating time showed the best gas-separation performance with CO_2_ permeance of 51 GPU and CO_2_/N_2_ ideal selectivity of 21.

## 1. Introduction

CO_2_ emission is inevitable owing to the growth of fossil fuel power plants and energy-intensive industries [1]. The excess of CO_2_ emission has significantly affected the global warming, sea level rise, and climate changes. Therefore, the conversion, capture, and separation of CO_2_ are crucial to tackle the abovementioned environmental problems attracting a plenty of attention in science and engineering in 21st century [2,3,4]. Several conventional techniques like amine adsorption, Pressure Swing Adsorption (PSA), and cryogenic distillation are used for CO_2_ separation [5,6,7]. However, these processes are energy and cost intensive. Membrane process is an energy efficient technology for CO_2_ separation [8]. The characteristics of the membrane process, such as simple operation, small footprint, and low cost, make it more competitive than the conventional gas-separation processes [9].

Both flat sheet and hollow-fiber membranes can be applied for CO_2_ separation [8,10,11]. Comparing these two types of membrane configuration, it can be stated, that hollow fibers are easier to be scaled-up, owing to their high packing density and a self-supporting structure [8,12,13,14]. Polymer materials such as polysulfone (PSf) [15,16,17], polyetherimide (PEI) [18,19,20,21], polyimide (PI) [22,23], polyacrylonitrile (PAN) [24], and polyvinylidene fluoride (PVDF) [25] are commonly applied for the preparation of hollow fibers.

Membranes offering the high gas permeance are crucial to the gas-separation process at industrial scale. To obtain a membrane with high gas permeance and reasonable selectivity, either the highly permeable materials are used or the thickness of dense selective layer must be reduced. However, highly permeable materials show rather low selectivity, due to the trade-off relationship of polymeric membranes [26]. Therefore, the preparation of asymmetric composite membranes consisting of a thin dense layer and a porous substrate is a desirable method to improve the gas-separation performance of membranes [27].

The dip-coating technique [28,29,30] and interfacial polymerization (IP) [31,32,33] are generally applied for the preparation of asymmetric composite membranes. The formation parameters play important roles in the formation of composite membrane with high gas-separation performance. Madaeni et al. [28] prepared polydimethysiloxane (PDMS) coated polyethersulfone (PES) composite membranes for CO_2_ capture. They investigated the coating conditions such as coating temperature, PDMS concentration and the number of sequential coatings. A total of 5 wt% PDMS was reported as an optimal concentration for the dip-coating process. The increase of the coating layer number increased the PDMS layer thickness and selectivity, however, significantly decreased the gas permeance [28]. Li et al. [34] prepared PDMS/polyacrylonitrile (PAN) composite hollow-fiber membranes via the dip-coating method for CO_2_/N_2_ separation. It was found that the pre-wetting of PAN substrate could inhibit the intrusion of PDMS enabling the formation of defect-free selective layer [34]. Chen et al. [35] fabricated polyether block amide (Pebax)/PDMS/PAN composite hollow-fiber membranes via the dip-coating method and demonstrated that the prepared membranes could be used for flue gas treatment and hydrogen purification. PDMS was firstly coated on the PAN substrate to act as a gutter layer. The Pebax solution intrusion was minimized due to the PDMS gutter layer. Consequently, high gas permeance was obtained. The coating time and coating solution concentration are very important to the preparation of defect-free multi-layer hollow-fiber membranes [35]. Jo et al. [36] prepared inside coated thin film composite hollow-fiber membranes via interfacial polymerization. It was found that the concentrations of amine solution and acid chloride solution play crucial roles in determining the morphology and gas transport behavior of membranes. The high CO_2_/CH_4_ selectivity of the prepared membrane was attributed to the formation of ultrathin film and the properties of binary amino groups [36]. The aforementioned examples demonstrate the need for the creation of thin dense layer on the porous supports for gas separation. The conditions for the preparation of dense thin layer on porous supports are specifically addressed, since the optimization of the fabrication conditions is critically important to the formation of defect-free thin layer. These results from the literature are very close to our research work, since they present the gas-separation performance of PDMS or Pebax layer on various polymer supports.

In addition to the experimental investigation on the gas-separation behavior in the thin-film-composite hollow-fiber membranes, the theoretical and modeling studies are also applied for the study of mass transfer through hollow-fiber membranes in literature. Xiao et al. [37] applied a fractal model for the capillary flow through a single tortuous capillary with rough surfaces in fibrous porous media. Ghobadi et al. [38] conducted a 2D mass-transfer simulation model, using computational fluid dynamics (CFD) for separation of CO_2_ from a binary gas mixture of CO_2_/CH_4_ by means of polytetrafluoroethylene (PTFE) hollow-fiber membrane contactor.

PDMS and PEI are commercially available polymers. PDMS has been widely used in gas-separation process. The aim of this research was to prepare PDMS/PEI composite hollow fiber membranes via the dip-coating method for CO_2_/N_2_ separation. The influences of coating conditions such as the concentration of coating solution, coating time, curing temperature and the number of coating layers on the membrane structure, the gas permeance and ideal selectivity were systematically investigated. The optimization of coating conditions addressed in this study is one of the most outstanding research issues for the fabrication of defect-free thin dense layer on the porous support for gas separation. This experimental work is oriented to the practical application instead of theoretical and modeling studies even though they are also very important to the membrane-based gas-separation processes.

## 2. Experimental

### 2.1. Materials

Polyetherimide (PEI, Ultem 1000) pellets were kindly provided by Membrain (Stráž pod Ralskem, Czech Republic). *N*-methyl-2-pyrrolidone (NMP, 99.5%) was bought from Linegal Chemicals Sp. z o.o. (Warsaw, Poland). Methanol and n-hexane were purchased from Alchem Grupa Sp. z o.o. (Toruń, Poland). Pure CO_2_ (99.999%) and N_2_ (99.999%) gases were purchased from Air Products Sp. z o.o. (Siewierz, Poland). The fast solidified epoxy resin Araldite 2000 and 3M EPX Quadro Mixing Nozzles were delivered by Farnell (Warsaw, Poland). Elastosil LR 6240A (containing platinum catalyst) and Elastosil LR 6240B (containing crosslinker) were provided by Wacker Chemie AG Polska Sp. z o.o. (Warsaw, Poland).

### 2.2. Fabrication of PEI Hollow Fibers

Dope solution with PEI concentration 20 wt% was prepared by dissolving PEI pellets in NMP in a round bottom flask under refluxing condition at 60 °C for 24 h. Prior to dissolving PEI pellets in NMP, they were dried in oven at 100 °C to remove the traces of moisture. The prepared dope solution was transferred into a laboratory screw cap bottle and left for 24 h for degassing. The PEI hollow fibers were prepared via the dry-jet wet spinning process by using a laboratory-built spinning system [8,39]. The spinning conditions are shown in Table 1. In the spinning process, gear pump was used to deliver the dope solution at a specific extrusion rate from the stainless-steel reservoir to a spinneret. The bore fluid was delivered into the spinneret simultaneously by using a syringe pump. The as-spun hollow fibers went through an air gap and free fall into a coagulation bath containing distilled water, at room temperature. The prepared hollow fibers were cut and soaked in another water bath for 2 days, to remove the remaining NMP solvent. The hollow fibers from water bath were immersed into methanol for 12 h. Afterwards, the methanol-wet hollow fibers were immersed into hexane for 12 h. At last, hollow fibers were taken out from hexane and dried at room temperature, before further investigations. The physical properties of PEI hollow fibers have been fully investigated and presented in our previous work [39]; the basic parameters related to the diameters, wall thickness, skin layer thinness, and gas transport properties of hollow fibers are gathered in Table 2.

### 2.3. Dip-Coating Procedure

The coating process was performed on the outer surface of membranes, by applying the following procedure. First of all, PDMS component A and B with mass ratio of 1:10 were dissolved in hexane, to prepare 1.5, 3, 5, 7, 10, 15, and 20 wt% PDMS-coating solution. The solution was prepared by stirring its components for 2 h at room temperature. The PEI hollow fibers were immersed into the PDMS solutions of various concentrations for 10 min at room temperature. To investigate the coating time effect, hollow fibers were immersed into 15 wt% PDMS solution for various coating time of 0.5, 1, 3, 5, 7, 10, and 15 min at room temperature. The PDMS/PEI membranes prepared from 15 wt% PDMS solution and 10 min coating time were cured at various temperatures of 25, 50, 80, and 130 °C for 1 h. Moreover, a sequential coating process was conducted by dip-coating hollow fibers into 15 wt% PDMS solution for 10 min, several times, at room temperature, to investigate the effect of the number of PDMS layers. All the prepared PDMS/PEI composite hollow-fiber membranes were dried in air for at least 48 h, to remove the solvent and to fully cure the PDMS.

### 2.4. Characterization of PDMS/PEI Membranes

The morphology of the fabricated PEI hollow fibers and PDMS/PEI composite membranes were characterized by using Scanning Electron Microscope (SEM)—LEO 1430 VP microscope (Leo Electron Microscopy Ltd., Cambridge, UK). The scanning was performed at an accelerating voltage of 30 keV. To analyze the cross section of hollow fibers, the samples were prepared by fracturing fibers frozen in liquid nitrogen. Prior to the analysis, samples were sputtered with a conductive layer (thickness in the range of 2–6 nm) of Au/Pd (80/20) alloy. The energy-dispersive X-ray spectroscopy (EDX) analysis was conducted by using Phenom Prox/Pro/Pure, Generation 5 (Phenom-Word B. V., Eindhoven, The Netherlands). The PDMS layer thicknesses taken at the top and bottom parts of the prepared composite membranes were measured using SEM photos and ImageJ software (University of Wisconsin, Madison, WI, USA).

### 2.5. Module Preparation and Gas Permeance Measurements

To prepare the module, 2 hollow fibers with a length of 15–20 cm were assembled as a bundle. One hollow-fiber bundle was placed in a glass tube. Briefly, the single hollow-fiber bundle was placed in a glass tube. Both ends of the glass tube were sealed with a 5 min fast solidified epoxy resin (Araldite, Winterthur, Switzerland). Subsequently, one end of the glass tube was opened by using a scalpel before the complete solidification of epoxy resin. The details related to the module preparation are described elsewhere [39]. Pure N_2_ and CO_2_ were used for the single gas permeance tests. The trans-membrane pressure was set at 2 bar for all measurements, at room temperature, i.e., 25 °C. To ensure the accuracy of experiments, the gas permeance measurements were accomplished 3 times, in the stabilized conditions. A bubble flow meter was used to measure the gas flow rate. The permeances of gases expressed in GPU and the ideal selectivity expressed in the ratio of CO_2_ permeance to N_2_ permeance were used to estimate the gas-separation performance of PDMS/PEI composite hollow-fiber membranes. The permeances (P/d), of gases through the hollow-fiber module were calculated by using Equation (1):

(1) $$\frac{P}{d}=\frac{Q}{\Delta \mathrm{pA}}=\frac{Q}{2n\pi \mathrm{rl}\Delta p}$$

where P is the permeability (Barrer), d is the thickness of membrane selective layer (cm), Q is the flux of gas permeation rate (cm^3^ (STP)/s), Δp is the pressure difference across the membrane (cmHg), A is the effective membrane area (cm^2^), n is the number of hollow fibers, r is the outer radius (cm) of hollow fiber, and P/d is the gas permeance expressed in GPU (1 GPU = 10^−6^ cm^3^ (STP) cm^−2^ s^−1^ cmHg^−1^).

The ideal selectivity, α, is defined as the permeability coefficients or permeances ratio of two pure gases (Equation (2)):

(2) $$\alpha_{12}=\;\frac{{\left(P/d\right)}_{1}}{{\left(P/d\right)}_{2}}=\frac{P_{1}}{P_{2}}$$

## 3. Results and Discussion

### 3.1. Membrane Morphology

**Figure 1:**
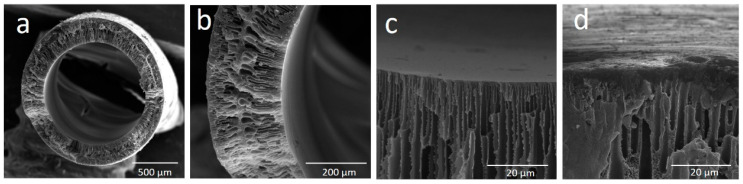
SEM pictures of uncoated PEI hollow fibers: (**a**) overall cross-section, (**b**) enlarged cross-section, (**c**) inner side, and (**d**) outer side.

The structure and morphology of the cross-section of the uncoated PEI hollow fibers are shown in Figure 1. Figure 1a presents the overall cross-section of the prepared hollow fiber. The prepared hollow fibers possess finger-like macrovoids near lumen and shell sides, tear-like macrovoids underneath the finger-like macrovoids, microporous structure in the middle of hollow-fiber wall (Figure 1b), and relatively thin dense skin layer on the inner surface (Figure 1c) and outer surface (Figure 1d) of hollow fibers. The resulting membrane morphology is affected by the polymer–solvent interactions, solvent–coagulant interactions and the concentration and viscosity of the dope medium [40,41]. The formation of finger-like and tear-like macrovoids in hollow fibers can be attributed to the water intrusion and the fast mass exchange between the solvent in the polymer solution and water (non-solvent), since distilled water was used as bore fluid and outer coagulant in the spinning process. Water is a strong non-solvent, while NMP has weaker interaction towards PEI; hence, it formed instant phase de-mixing, which created the finger-like and tear-like macrovoids [42]. The finger-like macrovoids near the shell side were shorter than the ones near the lumen side (Figure 1b). This is because of the formation of thin skin dense layer on the outer surface which impeded the water intrusion process. In the spinning process, the as-spun hollow fiber went through an air-gap distance of 25 cm (Table 1), and then to the water coagulant bath; the solvent evaporated during this short time, which increased the viscosity of polymer solution near shell side. As a result, the thin dense skin layer was formed and the solvent/non-solvent exchange process was slowed down.

**Figure 2:**
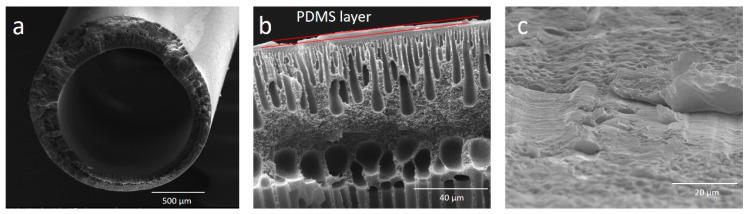
SEM pictures of polydimethysiloxane (PDMS)/PEI membrane prepared from 15 wt% PDMS solution at 10 min coating time: (**a**) overall cross-section, (**b**) enlarged cross-section, and (**c**) outer PDMS coating layer.

Figure 2 shows the morphology of PDMS/PEI membranes prepared by using the dip-coating method. The overall cross-section of PDMS/PEI membrane was shown in Figure 2a. It is observed that a thin PDMS selective layer was formed on the outer surface of PEI hollow fibers (Figure 2b,c).

**Figure 3:**
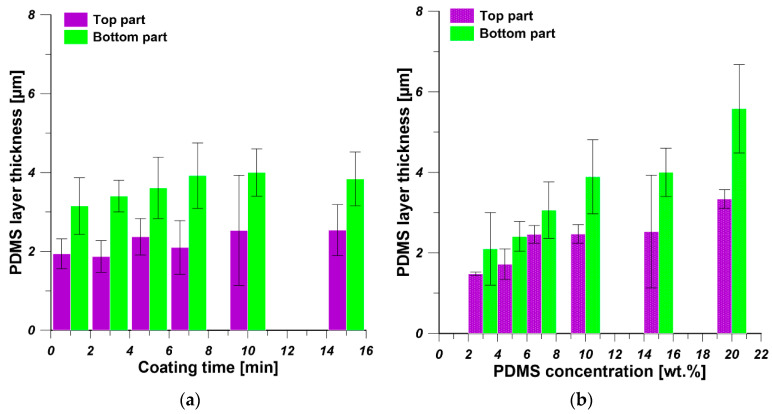
The thickness of PDMS selective layer: (**a**) the effect of coating time at constant concentration of PDMS solution equal to 15 wt%; (**b**) the effect of PDMS concentration at constant coating time equal to 10 min.

As it is shown in Figure 3, the coating time and the coating solution concentration affect the thickness of PDMS layer. When 15 wt% of PDMS solution was used, the thickness of PDMS layer at the bottom part of PDMS/PEI composite membrane increased from 3 to 4 µm, along with the change of coating time from 1 to 7 min. The further increase in coating time to 15 min did not change the thickness of PDMS layer (Figure 3a). The thickness of PDMS layer at the top part of PDMS/PEI composite membrane slightly increased from 1.9 to 2.5 µm, accompanied by the change of coating time from 1 to 15 min (Figure 3a). With an increase in coating time, more PDMS chains could diffuse and adhere onto the substrate surface and reach a stable state. Therefore, the PDMS layer thickness increased when the coating time increased from 1 to 7 min, while it became constant with the further increase of coating time to 15 min. The coating time has a more significant influence on the PDMS layer thickness at the bottom part than the top part of PDMS/PEI composite membrane. When the coating time is set as 10 min, the thickness of PDMS layer at the top part and the bottom part of PDMS/PEI composite membrane increased from 1.5 and 2 µm to 3.3 and 5.6 µm, along with the growth of PDMS concentration from 3 to 20 wt%, respectively (Figure 3b). According to the Landau–Levich theory, the thickness of coating layer is positively proportional to the viscosity of coating solutions [43]. The viscosity of PDMS solution increased with the concentration increase of PDMS solution. As a result, the thickness of the PDMS layer augmented with an increase in PDMS concentration. It was reported in the literature that the thickness of the coating layer increased with the increase in the concentration of coating solution and coating time [16,28,44,45]. When comparing Figure 3a,b, it is found that the concentration of PDMS solution predominantly affected the thickness of PDMS selective layer. Furthermore, the thickness of PDMS layer at the bottom part is slightly higher than the thickness of PDMS layer at the top part. In the dip-coating process, the interplay of several parameters, e.g., viscous force, solvent evaporation and draining, surface tension, gravity, and hydrodynamic factors in the the layer deposition region, governs the layer thickness and the position of the drying front. The bottom part was always close to the solution reservoir, while the top part was relatively far from the coating solution. A wet film with higher concentration was formed at the drying front, due to the higher ratio of surface area to volume, which results in the drawing of solution from surrounding area. When a dry film was formed at the drying front, a capillary force appeared on the solution, resulting in the thickening of the coated film [46,47,48,49].

**Figure 4:**
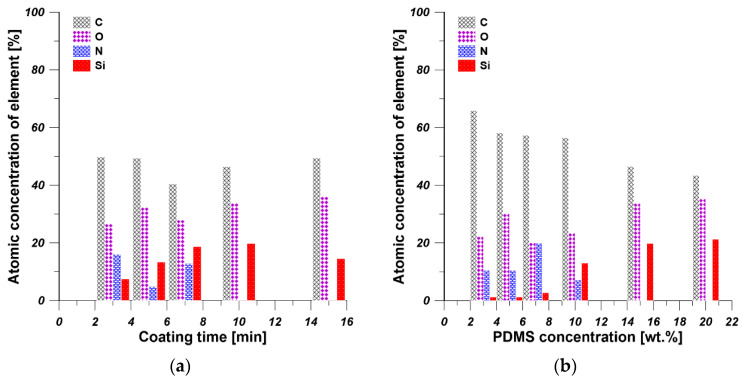
EDX surface mapping results for PDMS/PEI membranes fabricated (**a**) by changing the coating time, using PDMS concentration equal to 15 wt%; (**b**) by using various PDMS concentrations at constant coating time equal to 10 min.

Figure 4 shows the EDX results of PDMS/PEI membranes prepared at various coating times and using various concentrations of PDMS solution. The detection of carbon, oxygen, and nitrogen can be attributed to the characteristics of PEI, which contains imide group in its chemical structure. The detection of silicon element indicates the PDMS was successfully coated on the outer surface of substrate. As it is shown in Figure 4a, with an increase in coating time, the atomic concentration of nitrogen and silicon decreased and increased, respectively, which indicates the more desirable formation of PDMS selective layer. When the coating time is over 10 min, a dense PDMS layer completely covered the outer surface of support. These results are consistent with the results of gas permeance of PDMS/PEI membranes prepared at various coating time (Section 3.2.1). As the EDX results in Figure 4b show, the atomic concentration of silicon increased significantly from 1.26% to 21.31%, along with the change of coating solution from 3 to 20 wt%. These results are also consistent with the results of PDMS layer thickness (Figure 3b) and gas permeance of PDMS/PEI membranes prepared from various concentrations of PDMS solution (Section 3.2.2). As discussed above, the silicon concentration increased with the growth of coating time and PDMS concentration, which is due to the formation of thicker PDMS layer and deposition of more PDMS chains on the support.

### 3.2. Gas-Separation Performance

The prepared unmodified PEI hollow fibers were highly permeable to CO_2_ and N_2_, with the ideal selectivity close to 1. To obtain a PDMS/PEI composite hollow-fiber membrane with high selectivity and reasonable gas permeance via the dip-coating method, the optimization of coating conditions plays a crucial role. The influence of the chosen coating conditions on the gas-separation performance of PDMS/PEI composite hollow-fiber membrane is discussed in this section.

#### 3.2.1. The Effect of Coating Time

**Figure 5:**
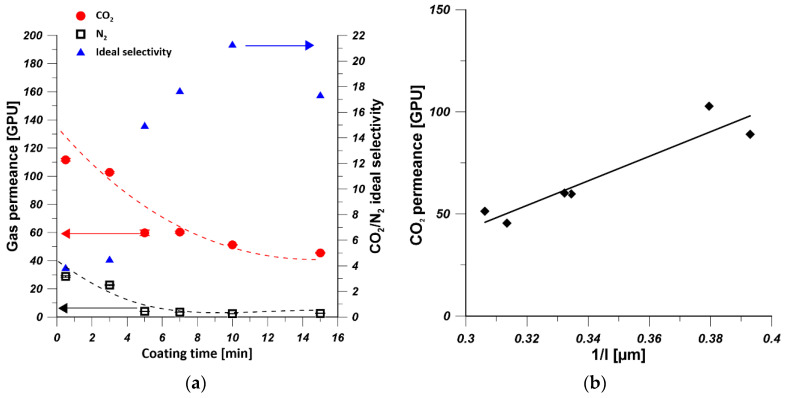
(**a**) The effect of coating time on gas permeance and ideal selectivity of PDMS/PEI membranes prepared from 15 wt% of PDMS solution (the dashed line indicates only the trend of gas permeance change). (**b**) The influence of PDMS layer thickness (l) on CO_2_ permeance of PDMS/PEI membranes prepared from various 15 wt% of PDMS solution at various coating time (the average thickness of PDMS layer was calculated based on the thicknesses of PDMS layer at the top and bottom parts).

To investigate the influence of coating time on the gas permeance, PDMS/PEI composite membranes were fabricated by immersing PEI supports in 15 wt% of PDMS solution at various time from 0.5 to 15 min and cured at 25 °C. Figure 5a shows the trends of CO_2_ and N_2_ permeances, and the ideal selectivity of PDMS/PEI membranes prepared at various coating times. It can be realized that the CO_2_ and N_2_ permeances decreased significantly when the coating time changed from 0.5 to 5 min. Then the CO_2_ permeance continued to decrease slightly, while the N_2_ permeance leveled off for the coating time in the range of 5 to 15 min. This is because the kinetic diameter of N_2_ molecule (0.36 nm) is larger than that of CO_2_ molecule (0.33 nm). Moreover, the CO_2_ molecules possess higher affinity to PDMS [50]. Hence, CO_2_ molecules are transported much faster than N_2_ ones, and they are more sensitive to the thickness change of PDMS layer (Figure 3a). On the other hand, the CO_2_/N_2_ ideal selectivity increased to the maximal values of 21, when the coating time approached 10 min. Afterwards, the CO_2_/N_2_ ideal selectivity decreased only slightly. This is caused by a fact that with an increase of coating time, the defect-free PDMS layer was formed, resulting in the increased value of the ideal selectivity. However, the coated PDMS might be re-dissolved in the solvent when a longer coating time is applied. As a result, defects might be formed and the selectivity was reduced. It is found that 10 min was the optimal coating time for the preparation of PDMS/PEI membranes with a high gas-separation performance. Similar results were also found by Chen et al. [35]. The thickness of PDMS layer of PDMS/PEI composite membrane prepared from 15 wt% of PDMS solution at various coating times was measured. Figure 5b shows the influence of PDMS layer thickness on CO_2_ permeance; it can be seen that CO_2_ permeance drops with the formation of thicker PDMS layer due to the increased mass transfer resistance [51,52]. According to the solution-diffusion model, the gas permeance is inversely proportional to the thickness of selective layer [53]. It can be seen in Figure 5b that the CO_2_ permeance is linearly proportional to the reciprocal of PDMS layer thickness, which revealed the hypothesis that the gas transport through the PDMS layer obeys the solution-diffusion mechanism.

#### 3.2.2. The Effect of PDMS Concentration

**Figure 6:**
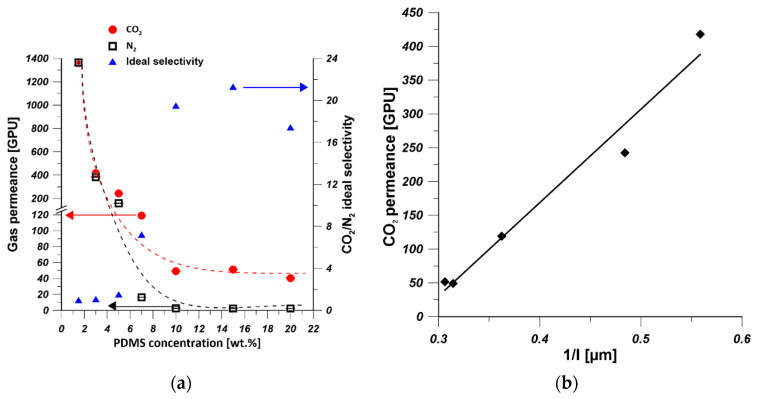
(**a**) The effect of PDMS concentration on gas permeance and ideal selectivity of PDMS/PEI membranes prepared at coating time of 10 min (the dashed line only indicates the trend of gas permeance change). (**b**) The influence of PDMS layer thickness on CO_2_ permeance of PDMS/PEI membranes prepared from various PDMS concentrations, at a coating time of 10 min (the average thickness of PDMS layer was calculated based on the thickness of PDMS layer at the top and bottom parts).

As it is shown in Figure 6a, the CO_2_ permeance and N_2_ permeance dramatically decreased from 1360 GPU to 49 GPU and 2.5 GPU, respectively, when the PDMS concentration changed from 1.5 wt% to 10 wt%. Then the CO_2_ permeance was constant at 50 GPU but slightly decreased to 40 GPU with the change of PDMS concentration from 15 wt% to 20 wt%. However, N_2_ permeance was leveled off at 2.4 GPU when the PDMS concentration was between 10 and 20 wt%. The decrease in gas permeance was attributed to the increase in PDMS layer thickness (Figure 3b). The CO_2_/N_2_ ideal selectivity increased to the maximal values of 21, when the PDMS concentration increased to 15 wt%. However, it decreased when 20 wt% PDMS solution was used for coating. When the PDMS solutions of 1.5, 3, 5, and 7 wt% were used as coating solution, defects might be formed on the PDMS selective layer. These findings are consistent with the EDX mapping results (Figure 4b). When 20 wt% of PDMS solution was used as coating solution, the prepared composite hollow-fiber membrane possessed the lowest CO_2_ permeance owing to the formation of a thicker PDMS selective layer (Figure 3b). The decrease of CO_2_/N_2_ ideal selectivity can be explained by the relatively larger decrease in CO_2_ permeance comparing to the N_2_ permeance which resulted from the sensitivity of CO_2_ to the change of PDMS layer thickness. Chong et al. [54] found similar results where PDMS/PSf composite hollow-fiber membranes experienced lower O_2_ permeance and O_2_/N_2_ selectivity due to the formation of thicker PDMS layer which increases the mass transfer resistance. The thickness of PDMS layer of PDMS/PEI composite membrane prepared from various concentrations of PDMS solution at coating time equal to 10 min was measured. As it is shown in Figure 6b, CO_2_ permeance is linearly proportional to the reciprocal of PDMS layer thickness which is in good agreement with solution-diffusion model [53]. The decrease in CO_2_ permeance was attributed to an increase in the thickness of PDMS selective layer [51,53].

#### 3.2.3. The Effect of the Curing Temperature

**Figure 7:**
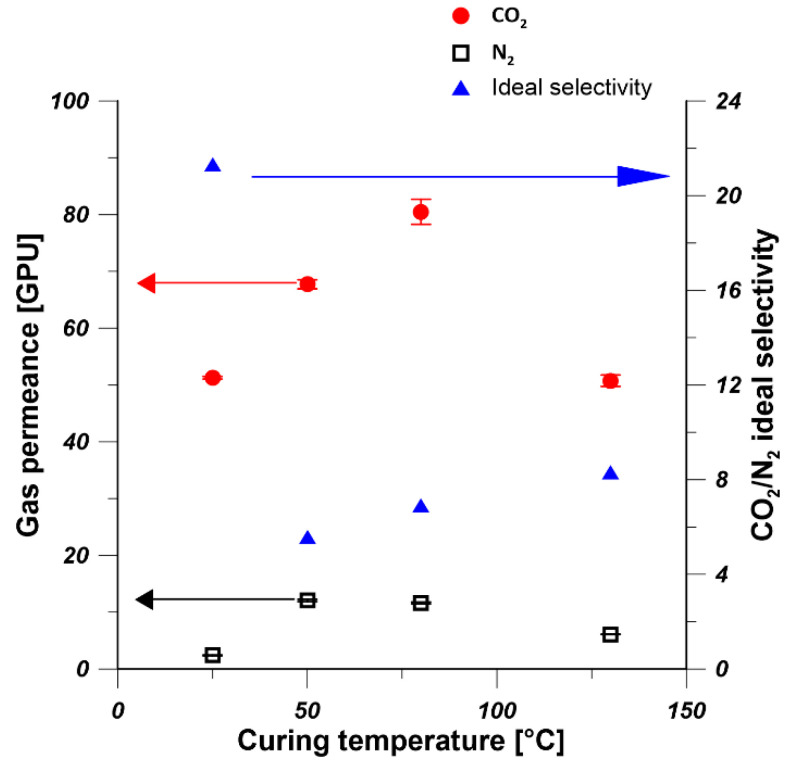
Effect of curing temperature on gas permeance and ideal selectivity of PDMS/PEI membranes (PDMS concentration—15 wt% and coating time—10 min).

To investigate the effect of curing temperature on the gas permeance of PDMS/PEI membranes, 15 wt% of PDMS solution was coated on PEI hollow-fiber substrate for 10 min. Subsequently, the coated hollow fibers were transferred to an oven and crosslinked at various temperatures, i.e., 25, 50, 80, and 130 °C. As it is shown in Figure 7, the gas permeances of CO_2_ and N_2_ experienced an increase and decrease trend with the increase in annealing temperature. The PDMS/PEI composite hollow-fiber membranes cured at 25 °C showed the highest CO_2_/N_2_ selectivity. Therefore, 25 °C was used as the curing temperature and membranes were annealed in air at 25 °C. Madaeni et al. [28] observed that the CO_2_ permeance and CO_2_/N_2_ selectivity of PDMS/PES flat sheet membrane decreased slightly with the increase in curing temperature from 25 °C to 200 °C. Kargari et al. [21] found that the curing temperature has little effect on the H_2_ and CH_4_ permeances and selectivity of PDMS/PEI flat sheet membranes. It can be seen that higher curing temperature could not enhance the gas-separation performance of PDMS coated composite membranes. This is because the viscosity of coating solution decreased with the increase in curing temperature, which resulted in the weakened adhesion between PDMS chain and substrate and the formation of undesirable defects [43].

#### 3.2.4. The Effect of the Multiple Coating of PDMS

**Figure 8:**
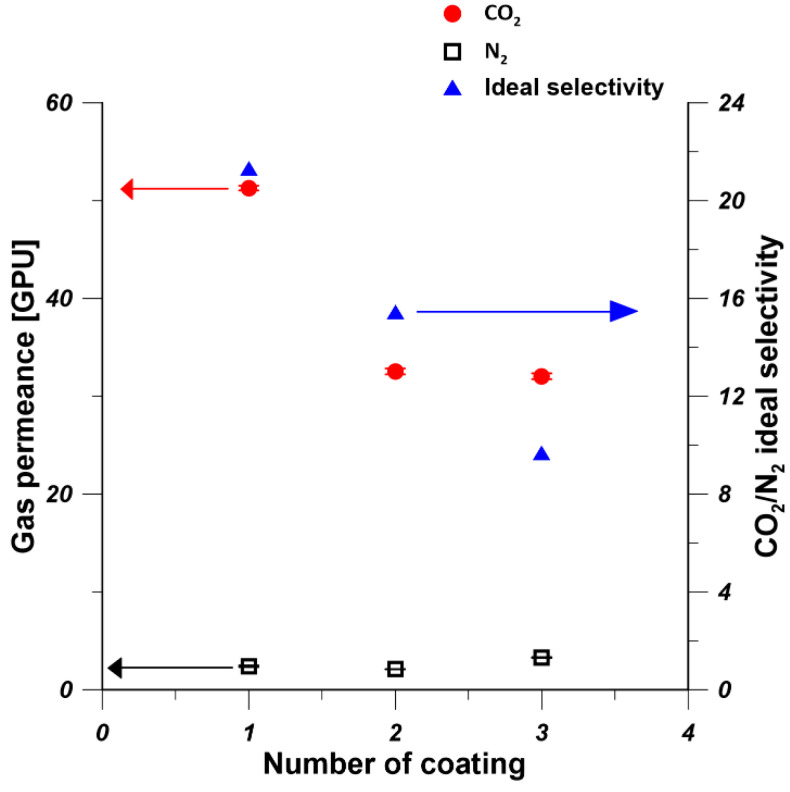
Effect of the number of coating layers on gas permeance and ideal selectivity of PDMS/PEI membranes (PDMS concentration—15 wt% and coating time—10 min).

To investigate the influence of the multiple coating of PDMS layer on the gas permeance of PDMS/PEI membranes, one, two, and three PDMS layers were coated on the hollow-fiber substrate by using 15 wt% PDMS solution for 10 min at 25 °C. As it is shown in Figure 8, when the PDMS coating changed from one time to three times, the N_2_ permeance was practically constant, while the CO_2_ permeance decreased due to the increase in the thickness of PDMS layer from 3.3 to 5.5 µm. Therefore, CO_2_/N_2_ ideal selectivity decreased with the increase in the number of PDMS layers. Similar findings were presented in the work of Selyanchyn et al. [52]. They prepared Pebax/PDMS/porous support thin-film composite flat-sheet membranes, where a PDMS layer was used as a gutter layer and a Pebax layer was used as the selective layer. It was reported that when the thickness of Pebax and PDMS layer increased from 0.29 and 2.3 μm to 0.39 and 10.8 μm, respectively, the CO_2_ permeance and CO_2_/N_2_ selectivity decreased from 300 GPU and 43 to 180 GPU and 35, respectively. The decrease in gas permeance is owing to the increase of thickness of selective layer. The increased thickness of selective layer imparted more significant influence on CO_2_ permeance that it on N_2_, which resulted in the decrease in CO_2_/N_2_ selectivity. This is because larger gas molecules are preferentially transported through PDMS selective layer [55]. However, Kargari et al. [21] found that when the number of coating layers increased, the H_2_ permeance decreased, while the H_2_/CH_4_ selectivity increased. This is because the defect-free coating layer was developed as the number of coating layer increased, which finally led to a better performance.

## 4. Comparison of CO_2_ and N_2_ Separation with Literature Data

Table 3 summarizes the previously reported works in which thin-film composite membranes have been prepared for CO_2_/N_2_ separation. Pebax or PDMS solution with low concentration was generally used as coating solution for the formation of selective layer on porous substrate [34,56]. When the prepared membranes possessed high CO_2_ permeance, the CO_2_/N_2_ selectivity was low [24,34]. The prepared membranes in this work possess satisfying CO_2_/N_2_ separation performance in comparison with the examples from the literature [56], which indicates the PDMS/PEI membranes were successfully fabricated.

## 5. Conclusions

PDMS/PEI composite hollow fiber membranes were successfully fabricated by using the dip-coating method. The thickness of PDMS layer was influenced by the concentration of coating solution and the coating time. The increase in coating time and the concentration of PDMS solution resulted in the decrease in gas permeance, due to the formation of defect-free PDMS layer and the increase in its thickness. The relation between gas permeance and PDMS layer thickness followed the solution-diffusion model. The increase in curing temperature and the number of coating layer imparted adverse effects on the gas-separation performance. The composite hollow fiber membrane prepared from 15 wt.% PDMS solution at 10 min coating time showed the best gas-separation performance with CO_2_ permeance of 51 GPU and CO_2_/N_2_ selectivity of 21. The gas-separation performance of PDMS/PEI composite hollow fiber membrane is comparable to literature ones.

The prepared PDMS/PEI composite hollow fiber membranes followed the trade-off relationship between permeance and selectivity [26]. To enhance the gas-separation performance by increasing the gas permeance and selectivity simultaneously, 2D nanomaterials, such as surface-modified graphene oxide (GO) containing CO_2_-philic functional groups [61,62,63] and nitrogen-doped graphene nanosheets [64,65], will be incorporated in the selective coating layer in the further works. Moreover, the conditions for the preparation of mixed matrix hollow fiber membranes [66] will be optimized by using chemometric methods [67].

## Figures and Tables

**Table 1:** Spinning parameters for polyetherimide (PEI) hollow-fiber fabrication.

Spinning Parameters	Spinning Conditions
Spinneret dimensions, outer diameter/inner diameter (mm/mm)	4.8/2.1
Bore fluid	Distilled water
Bore fluid flow rate (mL/min)	9–12
Bore fluid temperature (°C)	25 ± 2
Dry air gap length (cm)	25
Dope extrusion rate (mL/min)	7.6
Take up	Free fall
External coagulant	Water
Temperature of external coagulant (°C)	25 ± 2
Temperature of spinneret (°C)	25 ± 2

**Table 2:** Material and transport characteristics of the prepared hollow-fiber substrate [39].

d_outer_ (µm)	d_inner_ (µm)	l_wall_ (µm)	Outer Skin (µm)	Inner Skin (µm)	CO_2_ Permeance (GPU)	CO_2_/N_2_ Selectivity
1446 ± 100	1053 ± 81	194 ± 43	2.40 ± 0.90	0.37 ± 0.07	6427	1.07

**Table 3:** Comparison of the thin film composite hollow-fiber membranes for CO_2_/N_2_ separation (HF—hollow fiber and FT—flat sheet).

Membrane	Configuration	Pure Gas Permeance (GPU)		CO_2_/N_2_	Reference
		CO_2_	N_2_		
3 wt% PDMS/PAN	HF	2494	241	10.4	[34]
2 wt% PDMS/PAN	HF	2680	360	9	[34]
3 wt% PDMS/PSf	HF	55	1.56	35	[56]
3 wt% PDMS/PSf	HF	59	1.60	37	[56]
0.3 wt% PDMS/PAN	HF	5138	485	11	[24]
3 wt% PDMS/PSf	HF	200	6	33	[57]
3 wt% PDMS/PES-PI	HF	60	1.54	39	[58]
3 wt% PDMS/PSf	HF	64	2	32	[59]
PEBA/PDMS/PEI	FT	172	3.64	47	[29]
PDMS/PEBA/PDMS/PEI	FT	157	2.46	64	[29]
3 wt% Pebax/PSf	HF	23	0.64	36	[56]
3 wt% Pebax/PSf	HF	30	0.76	39	[56]
5 wt% Pebax/PEI	HF	48	2.00	24	[60]
15 wt% PDMS/PEI	HF	51	2.4	21	This work

## Data Availability

The data presented in this study are available on request from the corresponding author.
